# Liver and pancreatic fat fractions as predictors of disease severity in acute pancreatitis: an MRI IDEAL-IQ study

**DOI:** 10.1007/s00261-025-04809-y

**Published:** 2025-01-30

**Authors:** Kemal Panc, Hasan Gundogdu, Sumeyye Sekmen, Mustafa Basaran, Enes Gurun

**Affiliations:** 1 Karakoçan State Hospital, Elazığ, Turkey; 2 Samsun University, Samsun, Turkey; 3 Patnos State Hospital, Ağrı, Turkey

**Keywords:** Metabolic dysfunction–associated steatotic liver disease, Non-alcoholic fatty pancreatic disease, Acute pancreatitis, IDEAL, Proton density fat fraction

## Abstract

**Purpose:**

Metabolic dysfunction–associated steatotic liver disease (MASLD) and non-alcoholic fatty pancreatic disease (NAFPD) are metabolic diseases with rising incidence. Fatty infiltration may lead to dysfunction of the liver and pancreatic tissues. This study aims to quantify liver and pancreatic fat fractions and examine their correlation with disease severity in acute pancreatitis patients.

**Methods:**

The severity of acute pancreatitis was assessed using the revised Atlanta classification (RAC), computed tomography severity index (CTSI), and modified CTSI (mCTSI). Proton density fat fraction (PDFF) levels of the liver and pancreas were measured via IDEAL MRI. Patients were categorized into biliary and non-biliary pancreatitis groups. Correlations between PDFF levels and the RAC, CTSI, and mCTSI scores were analyzed.

**Results:**

A total of 127 patients were included, with MASLD present in 40.9% and NAFPD in 30%. Liver PDFF values were significantly higher in non-biliary pancreatitis (*p* = 0.040). Patients with MASLD exhibited higher CTSI and mCTSI scores (*p* = 0.009, *p* = 0.033, respectively). No significant differences were observed in severity scales between patients with and without NAFPD. Liver PDFF was positively correlated with CTSI and mCTSI scores in biliary pancreatitis. ROC analysis identified a liver PDFF > 3.9% (*p* = 0.002) and pancreatic corpus PDFF > 12.1% (0.028) as diagnostic markers for severe pancreatitis. In addition, a liver PDFF < 4.5% (*p* = 0.042) was an indicator for biliary pancreatitis.

**Conclusion:**

MASLD is associated with increased severity in acute pancreatitis. IDEAL MRI-derived PDFF levels of the liver and pancreas show potential in predicting severe acute pancreatitis and distinguishing between biliary and non-biliary etiologies.

**Supplementary Information:**

The online version contains supplementary material available at 10.1007/s00261-025-04809-y.

## Introduction

Acute pancreatitis (AP) is an acute inflammatory condition of the pancreas characterized by the destruction of acinar cells [1]. Clinically, AP is diagnosed based on characteristic abdominal pain, elevated serum amylase or lipase levels, and imaging findings. The most common etiological factors are gallstones and chronic alcohol consumption [2].

The Delphi consensus has recently proposed metabolic dysfunction–associated steatotic liver disease (MASLD) as a new terminology to replace non-alcoholic fatty liver disease, emphasizing its metabolic roots [3]. Both MASLD and non-alcoholic fatty pancreatic disease (NAFPD) share common risk factors with AP, including obesity, metabolic syndrome, and hyperlipidemia [4, 5]. The presence of MASLD has been shown to amplify inflammatory responses by increasing the production of proinflammatory cytokines, potentially exacerbating the severity of AP [6]. Intra-pancreatic fat accumulation is linked to pancreatic dysfunction and diseases such as acute pancreatitis, chronic pancreatitis, type 2 diabetes mellitus, and ductal adenocarcinoma [7]. During acute pancreatitis, pancreatic fat exerts a toxic effect on the parenchyma, causing localized injury known as peri-fat acinar necrosis, where tissue damage is most severe near necrotic fat and decreases with distance [8, 9].

Currently, liver biopsy is the preferred method for diagnosing and quantifying MASLD, but its limited sample size can lead to inaccurate assessments, especially in cases of focal fat accumulation [10]. Magnetic resonance spectroscopy remains the reference imaging technique for measuring liver fat, but samples only small liver regions [11]. The chemical shift imaging method, first described by Dixon in 1984, had confounding factors such as T1-related bias, T2* delay, and the spectral complexity of fat, which could lead to misleading results [12]. In 2005, Reeder et al. developed the IDEAL (Iterative Decomposition of Water and Fat with Echo Asymmetric and Least-squares Estimation) MRI technique, an advanced water-fat separation method that combines multiple gradient echoes at different echo times with a mathematical model to accurately quantify fat content from 0 to 100% [13]. Numerous studies have demonstrated a strong correlation between IDEAL MRI and both liver biopsy and magnetic resonance spectroscopy in assessing and grading liver fat content [14–16].

The results of this study may facilitate early risk stratification for severe acute pancreatitis, enabling more effective resource allocation, such as admission to intensive care. Moreover, accurate differentiation of biliary causes in borderline cases streamlines diagnostic workflows and guides targeted procedures, such as ERCP for choledocholithiasis. Finally, the study highlights the enhanced role of quantitative MRI in personalized patient care. This study aims to evaluate the effectiveness of IDEAL MRI in quantifying liver and pancreatic proton density fat fraction (PDFF) and to determine the correlation between these fat fractions and the clinical severity of acute pancreatitis based on Computed Tomography Severity Index (CTSI), modified CTSI (mCTSI), and the revised Atlanta classification.

## Material and methods

### Study design and patient selection

This retrospective study was approved by the local ethics committee (approval number: 2022/142), with informed consent waived due to its retrospective design and anonymized data. We included patients admitted with acute pancreatitis between November 2020 and December 2022 who met the following criteria: age ≥ 18 years, contrast-enhanced abdominal CT within the first 24 h, and abdominal magnetic resonance imaging (MRI)/magnetic resonance cholangiopancreatography (MRCP) with an IDEAL sequence within 48–72 h of admission. Exclusion criteria encompassed malignancy, pregnancy, acute or chronic hepatitis, missing data, and poor-quality CT/MRI images (e.g., severe pancreatic atrophy, fatty degeneration, artifacts). Additionally, alcoholic pancreatitis was excluded because its overlapping associations with both metabolic-and-alcohol related liver disease and alcohol-associated liver disease complicates data analysis.

The diagnosis of acute pancreatitis required two of the following three criteria: (1) typical abdominal pain, (2) amylase or lipase levels at least three times above normal, and (3) radiologic findings consistent with AP [17]. For effective management and follow-up, differentiating between biliary and non-biliary etiologies was essential. Biliary AP was defined by the presence of gallstones on imaging and/or elevated serum alanine aminotransferase (> 100 U/L) or total bilirubin (> 2.3 mg/dL) [18]. Cases not meeting these criteria were classified as non-biliary pancreatitis.

Laboratory results at admission, intensive care hospitalization, comorbidities (diabetes mellitus, chronic kidney disease, hypertension, coronary artery disease, history of malignancy, and history of prior pancreaticobiliary surgery) were recorded from the hospital information management system.

### Imaging protocol

Contrast-enhanced computed tomography (CT) examinations of the abdomen were performed using a 16-slice CT scanner (Toshiba Alexion Advance Edition 16, Japan). The scanning protocol included a pancreatic phase acquisition at 35–40 s post-contrast injection. Iodinated contrast medium was administered intravenously at a dose of 1 mL/kg body weight, with an injection rate of 4 mL/s. Imaging parameters were as follows: slice thickness, 1 mm; field of view (FOV), 30–40 cm; matrix, 512 × 512; pitch, 1; and tube voltage, 120 kVp. This protocol was designed to optimize visualization of pancreatic parenchyma and potential lesions during the peak enhancement phase.

Contrast-enhanced MRI/MRCP scans of the abdomen were performed on a 3-tesla MR unit (3T GE Discovery MR750w, GE Healthcare, Waukesha, Wisconsin, USA) using a 16-channel phased-array body coil. The imaging protocol comprised axial and coronal T2, axial T2 fat suppressed, diffusion-weighted imaging, coronal 3D MRCP, axial T1 LAVA with pre- and post-contrast dynamic images, as well as axial IDEAL IQ sequences for fat fraction mapping. Parameters for the IDEAL IQ sequence were set with TR of 6.9 msec, TE of 2.9 msec, ETL of 3, FOV ranging between 35 and 40 cm, matrix resolution of 160 × 160, bandwidth of 127 kHz, 8 mm slice thickness, and a 3-degree flip angle. As a gadolinium-based contrast agent, gadobutrol (Gadovist; Bayer Healthcare) was used at a standard dose of 0.1 mmol/kg.

### Image analysis

All analyses and measurements were performed independently of patient information by consensus between two radiologists with five and 10 years of experience. Pancreatic and peripancreatic findings, as well as local and systemic complications, were recorded and scored. Upon admission, the CTSI and mCTSI were calculated from CT images. [19, 20]. The CTSI combines the Balthazar grade (0: normal, 1: pancreatic enlargement, 2: pancreatic inflammation, 3: single peripancreatic fluid collection, 4: two or more fluid collections or gas in pancreas) and a pancreatic necrosis score (0: no necrosis, 2: <30%, 4: 30–50%, 6: >50% necrosis) for a total of up to 10 points. The mCTSI redistributes the scoring into pancreatic inflammation (0: normal, 2: intrinsic abnormalities, 4: fluid collection/fat necrosis), pancreatic necrosis (0: none, 2: <30%, 4: >30% necrosis), and extrapancreatic complications (0: absent, 2: present), also totaling up to 10 points. Both indices categorize severity as mild (CTSI: 0–3, mCTSI: 0–2), moderate (CTSI: 4–6, mCTSI: 4–6), or severe (CTSI: 7–10, mCTSI: 8–10).

Patients were categorized into mild, moderately severe, and severe acute pancreatitis based on the revised Atlanta classification (RAC) [21]. Mild acute pancreatitis (MAP) has no organ failure or complications. Moderately severe acute pancreatitis (MSAP) includes transient organ failure (resolving within 48 h), local complications, or exacerbation of comorbid diseases. Severe acute pancreatitis (SAP) involves persistent organ failure lasting over 48 h, affecting one or multiple organ systems. Organ failure was evaluated using the modified Marshall scoring system [22].

**Fig. 1:**
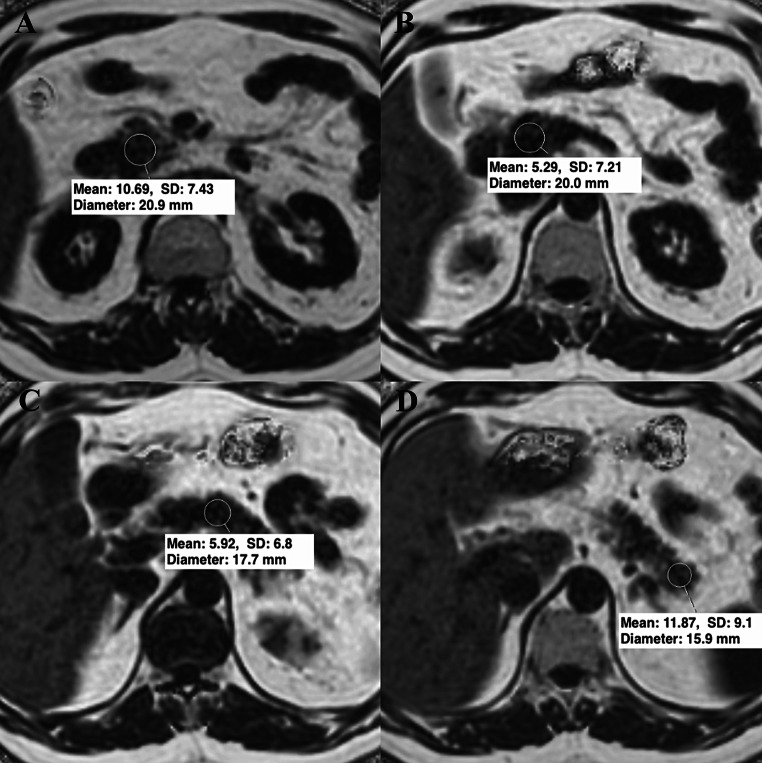
In a patient with a mean pancreatic PDFF level of 8.4%, placement of ROIs of appropriate size that do not overflow beyond the parenchyma; (**A**) Head, (**B**) Neck, (**C**) Body, (**D**) Tail

**Fig. 2:**
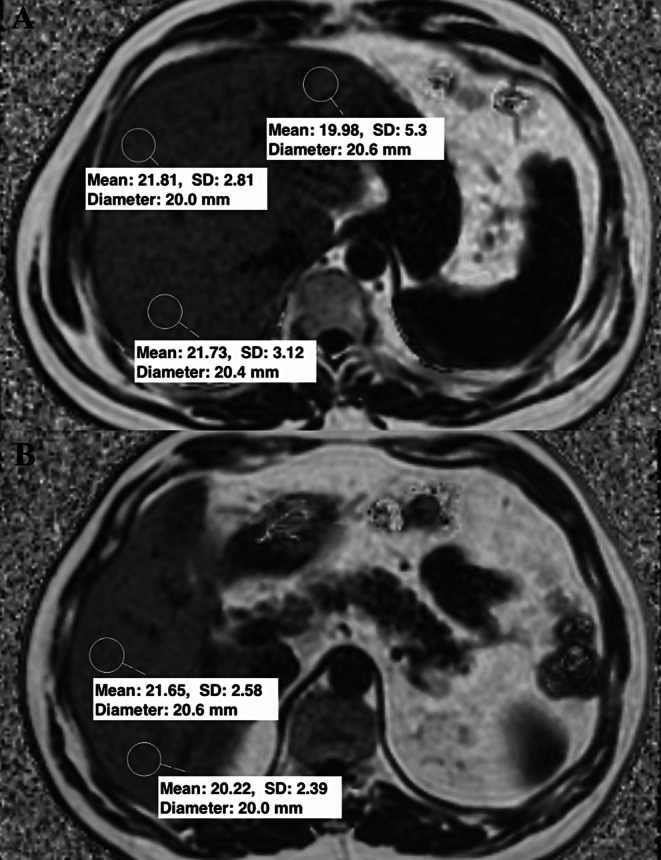
In a patient with a mean liver PDFF level of 21%, (**A**) ROIs of approximately 3 cm2 placed in segments 2, 7 and 8, (**B**) ROIs of similar size placed in segments 5 and 6

To measure pancreatic PDFF using the IDEAL Fat Fraction sequence, regions of interests (ROIs) were placed in the head, neck, body, and tail of the pancreas, ensuring inclusion of parenchyma without extending into surrounding adipose tissue or necrotic areas. In the context of necrosis, no measurements were taken from the affected region. The results were averaged (Fig. 1). The threshold PDFF level for diagnosing NAFPD was accepted as 10.4% [23]. To measure liver PDFF, ROIs of approximately 3 cm^2^ were placed in segments 2, 5, 6, 7, and 8 on the same sequence, avoiding bile ducts and major hepatic vessels (Fig. 2). The averaged liver PDFF used a threshold of 5% for diagnosing MASLD [24].

### Statistical analysis

All statistical analyses were performed using IBM SPSS Statistics software (version 22.0, Chicago, IL, USA). Descriptive statistics included counts, percentages, means with standard deviations (SD), and medians with ranges. Normality of variables was assessed using histogram plots, probability graphs, and tests such as Kolmogorov-Smirnov and Shapiro-Wilk. For normally distributed numerical variables, independent t-tests and one-way analysis of variance (ANOVA) were used for two-group and multiple-group comparisons, respectively. Non-parametric tests, such as the Mann-Whitney U and Kruskal-Wallis tests, were employed for non-normally distributed variables to reduce the risk of Type I errors. Nominal data were compared using Chi-square and Fisher’s exact tests. Correlations between variables were evaluated using Pearson’s correlation for normally distributed data and Spearman’s rank correlation for non-normally distributed data, providing both correlation coefficients and p-values to elucidate the strength and significance of associations. Receiver operating characteristic (ROC) analysis was used to assess the diagnostic performance of pancreatic and liver PDFF levels in predicting severe acute pancreatitis, calculating the area under the curve (AUC) with 95% confidence intervals (CI). Optimal cutoff values were determined using the Youden index, along with sensitivity, specificity, positive predictive value (PPV), and negative predictive value (NPV). A p-value of less than 0.05 was considered statistically significant. Additionally, post-hoc power analyses were performed for primary comparisons using two-tailed independent t-tests, assuming a medium effect size (Cohen’s d = 0.5) and an alpha level of 0.05, based on the observed sample sizes, to determine statistical power (1-β).

## Results

### Descriptive statistics

**Fig. 3:**
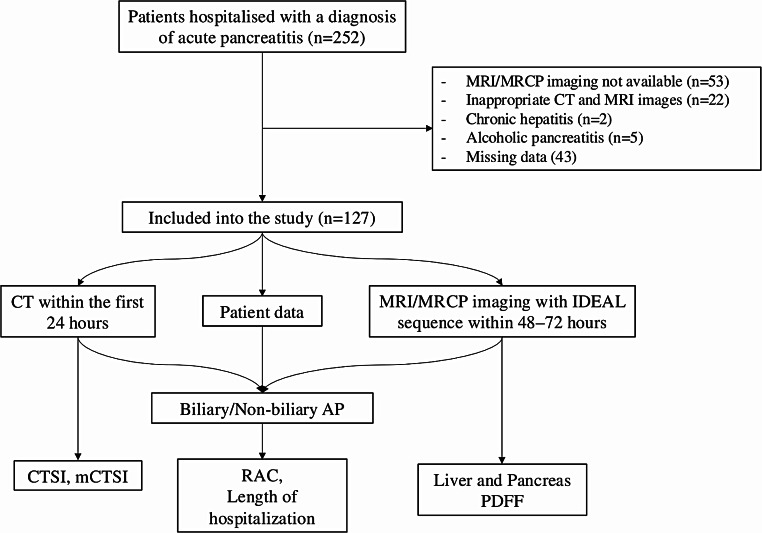
Patient selection and data acquisition process

The data of 252 patients were examined in a single-center study; 125 patients were excluded in accordance with the exclusion criteria, and 127 patients were included in this study (Fig. 3). The mean age of 61.7 years (± 17.0 years). The gender distribution was relatively balanced, comprising 48.0% males (*n* = 61) and 52.0% females (*n* = 66). Regarding the etiology of acute pancreatitis, the majority were classified as biliary pancreatitis (79.5%, *n* = 101), while non-biliary causes accounted for 20.5% of cases (*n* = 26).

The severity of acute pancreatitis was evaluated using both the CTSI and the mCTSI. According to the CTSI, 79.5% of patients were categorized as mild (*n* = 101), 19.7% as moderate (*n* = 25), and 0.8% as severe (*n* = 1). Similarly, the mCTSI classified 60.6% of patients as mild (*n* = 77), 37.8% as moderate (*n* = 48), and 1.6% as severe (*n* = 2). Based on the Revised Atlanta Classification, 63.8% of patients were diagnosed with mild (*n* = 81), 31.5% with moderately severe (*n* = 40), and 4.7% with severe acute pancreatitis (*n* = 6).

Patients were divided into two groups according to imaging findings: interstitial edematous pancreatitis and acute necrotizing pancreatitis. It was observed in 96.9% and 3.1% of cases, respectively. During follow-up for acute necrotizing pancreatitis, walled-off necrosis developed in 3 patients.

The mean PDFF for the pancreas was 9.0% ± 8.5, varying across different regions: head (8.6% ± 8.0), neck (8.2% ± 9.2), body (9.4% ± 9.3), and tail (9.9% ± 9.2). The liver had a mean PDFF of 5.9% ± 5.3.

### Post-hoc power analysis

To address potential concerns regarding the statistical power of our study, a post-hoc power analysis was conducted for key comparisons (Table 1). The comparisons between biliary and non-biliary pancreatitis, MASLD and non-MASLD patients, and NAFPD and non-NAFPD patients demonstrated adequate to high statistical power, reinforcing the robustness of these findings. In contrast, the comparison between mild acute pancreatitis and moderately severe to severe acute pancreatitis exhibited lower power.

**Table 1:** Post-hoc Power Analysis for key comparisons

Comparison	Effect Size (d)	Sample Sizes (*n*₁, *n*₂)	Power (1-β)	Interpretation
Biliary vs. Non-Biliary Pancreatitis	0.5	101, 26	0.82	Adequate power (> 0.80)
MASLD vs. Non-MASLD Patients	0.5	52, 75	0.90	High power (> 0.80)
NAFPD vs. Non-NAFPD Patients	0.5	38, 89	0.80	Adequate power (> 0.80)
MAP vs. MSAP to SAP Patients	0.5	81, 46	0.68	Below threshold, moderate risk of Type II errors

* *MASLD; Metabolic dysfunction–associated steatotic liver disease*,* NAFPD; Non-alcoholic fatty pancreatic disease*,* MAP; Mild acute pancreatitis*,* MSAP; Moderately severe acute pancreatitis*,* SAP; Severe acute pancreatitis)*

### Comparisons between groups

Liver PDFF was significantly higher in non-biliary pancreatitis (*p* = 0.040) and those with comorbidities (*p* = 0.025). There were no significant differences in pancreatic PDFF levels between the biliary and non-biliary AP groups. Pancreatic PDFF levels were significantly higher in patients with comorbidities (*p* = 0.001) and hypertension (*p* = 0.002) (Table 2).

**Table 2:** Distribution and analysis of pancreatic and liver PDFF levels based on the descriptive characteristics of the cases

Feature	Pancreas PDFF	*p*-value	Liver PDFF	*p*-value
	Median (min-max)		Median (min-max)	
Gender		0.447		0.567
Male	7.2 (0.9–44.6)		4.0 (1.0–27.0)	
Female	6.6 (0.8–50.2)		4.1 (0.9–22.5)	
Etiology		0.601		***0.040***
Non-biliary	6.4 (0.9–28.3)		5.6 (2.0–26.0)	
Biliary	7.0 (0.8–50.2)		3.9 (0.9–27.0)	
Comorbid disease		***0.001***		***0.025***
(+)	8.3 (1.7–50.2)		4.5 (0.9–27.0)	
(-)	4.0 (0.8–18.2)		3.4 (1.0-22.5)	
HT		***0.002***		0.117
(+)	8.1 (1.9–50.2)		4.5 (0.9–27.0)	
(-)	4.2 (0.8–27.0)		3.5 (1.0-22.5)	
DM		0.613		0.265
(+)	6.4 (1.7–22.8)		5.0 (1.5–26.0)	
(-)	7.2 (0.8–50.2)		4.0 (0.9–27.0)	
CAD		0.429		0.419
(+)	6.5 (2.6–44.6)		4.0 (1.0-22.5)	
(-)	7.2 (0.8–50.2)		4.0 (0.9–27.0)	
Pleural effusion		0.674		0.124
(+)	8.4 (1.9–18.2)		6.0 (2.0–26.0)	
(-)	6.7 (0.8–50.2)		4.0 (0.9–27.0)	

*HT; Hypertension,* DM; Diabetes Mellitus*,* CAD; Coronary Artery Disease*,* PDFF; Proton Density Fat Fraction*

** The Mann-Whitney U test was used in all analyses

Metabolic dysfunction–associated steatotic liver disease was identified in 52 (40.9%) patients. Patients with MASLD exhibited significantly higher CTSI scores (*p* = 0.009) and mCTSI scores (*p* = 0.033) compared to those without MASLD. Due to the small number of patients in the severe acute pancreatitis group, severe and moderate acute pancreatitis groups were merged in the RAC classification. However, no significant difference was found. Compared to patients without MASLD, those with MASLD exhibited a higher proportion of non-biliary etiologies (28.8% vs. 14.7%), although this difference was not statistically significant (*p* = 0.051) (Table 3).

**Table 3:** Comparison of AP severity and etiology in patients with and without MASLD

	Liver PDFF ≤ 5 (*n* = 75)	Liver PDFF > 5 (*n* = 52)	*p*-value
	Mean ± SD	Mean ± SD	
CTSI	2.1 ± 1.3	2.7 ± 1.2	***0.009*** ^***†***^
mCTSI	2.5 ± 1.4	3.1 ± 1.3	***0.033*** ^***†***^
Atlanta*			0.118^††^
MAP	52 (69.3)	29 (55.8)	
MSAP-SAP	23 (30.7)	23 (44.2)	
Etiology*			0.051^††^
Non-biliary	11 (14.7)	15 (28.8)	
Biliary	64 (85.3)	37 (71.2)	

^†^Independent samples t-test, ^††^Chi-square test, ^†††^Mann-Whitney U test

*N (%)

Non-alcoholic fatty pancreas disease was identified in 38 (30%) patients. In the comparison of patients with and without NAFPD, no significant differences were observed in the severity of acute pancreatitis as assessed by the CTSI (*p* = 0.494) and the mCTSI (*p* = 0.588). According to the RAC, there were no significant differences in the distribution of MAP and MSAP-SAP between patients with and without NAFPD (*p* = 0.367). Additionally, the etiology of acute pancreatitis, categorized as biliary or non-biliary, did not differ significantly between patients with and without NAFPD (*p* = 0.286).

Due to the small number of patients in the acute necrotizing pancreatitis, acute necrotizing collection, and walled-off necrosis groups, their relationships with liver and pancreas fat fractions could not be included in the analyses. When analysing patients with and without acute peripancreatic fluid collection, no significant difference was observed in PDFF levels in the pancreas (*p* = 0.748) or liver (*p* = 0.384).

### Correlation analyses

Correlations between age, CTSI and mCTSI and PDFF levels in pancreas and liver were analysed. A significant positive correlation was found between age and pancreatic PDFF (*p* < 0.001). CTSI (*p* = 0.002) and mCTSI (*p* = 0.003) exhibited a positive correlation with liver PDFF levels. When assessing according to etiology, a positive correlation was observed between liver PDFF and CTSI (*p* = 0.003) and mCTSI (*p* = 0.008) in the biliary pancreatitis but not in the non-biliary pancreatitis. However, there was no correlation between CTSI or mCTSI and pancreatic PDFF levels in both groups (Table 4).

**Table 4:** Correlation between pancreatic and liver PDFF levels and age, CTSI and mCTSI

Total (*n* = 127)		Pancreas PDFF	Liver PDFF
Age	rho	***0.457***	0.056
	p-value	***< 0.001***	0.533
CTSI	rho	0.098	***0.278***
	p-value	0.271	***0.002***
mCTSI	rho	0.010	***0.258***
	p-value	0.915	***0.003***
**Biliary pancreatitis** (*n* = 101)			
Age	rho	***0.482***	0.086
	p-value	***< 0.001***	0.394
CTSI	rho	0.174	***0.296***
	p-value	0.082	***0.003***
mCTSI	rho	0.091	***0.263***
	p-value	0.365	***0.008***
**Non-biliary pancreatitis** (*n* = 26)			
Age	rho	***0.399***	0.166
	p-value	***0.044***	0.417
CTSI	rho	-0.125	0.100
	p-value	0.541	0.627
mCTSI	rho	-0.252	0.150
	p-value	0.215	0.464

*PDFF; Proton density fat fraction,* CTSI; CT severity index*,* mCTSI; modified CT severity index*,* rho; Correlation coefficient*

** Spearman correlation test was used in all analyses

### ROC analyses

**Fig. 4:**
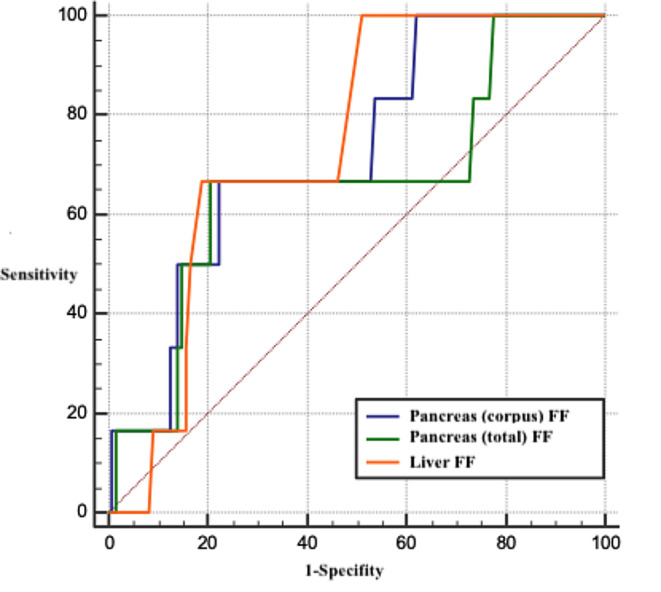
ROC curves of pancreatic and liver fat fraction values for the diagnosis of severe pancreatitis

The significance of pancreatic and liver fat fractions in severe pancreatitis, as determined by the RAC, was evaluated using ROC analysis. Both liver fat fraction (AUC = 0.74, *p* = 0.002) and pancreas corpus PDFF (AUC = 0.73, *p* = 0.028) were significantly associated with severe pancreatitis (Table 5)(Fig. 4). According to the Youden index, a pancreatic corpus fat fraction exceeding 12.1 demonstrated a sensitivity of 66.6%, specificity of 77.6%, positive predictive value of 12.9% and a negative predictive value of 97.9%. Similarly, a liver fat fraction above 3.9 exhibited a sensitivity of 100%, specificity of 48.7%, positive predictive value of 8.8% and a negative predictive value of 100%.

**Table 5:** ROC analyses of pancreatic and liver PDFF levels in severe pancreatitis

	AUC	%95 CI	*p*-value
Pancreas PDFF	0.66	0.575–0.745	0.228
Head PDFF	0.65	0.563–0.735	0.207
Neck PDFF	0.56	0.471–0.650	0.657
**Corpus PDFF**	0.73	0.640–0.801	***0.028***
Tail PDFF	0.64	0.547–0.720	0.403
**Liver PDFF**	0.74	0.655–0.814	***0.002***

*PDFF; Proton density fat fraction,* AUC; Area under the curve*,* CI; Confidence interval*

** Severe pancreatitis was determined according to the RAC

**Fig. 5:**
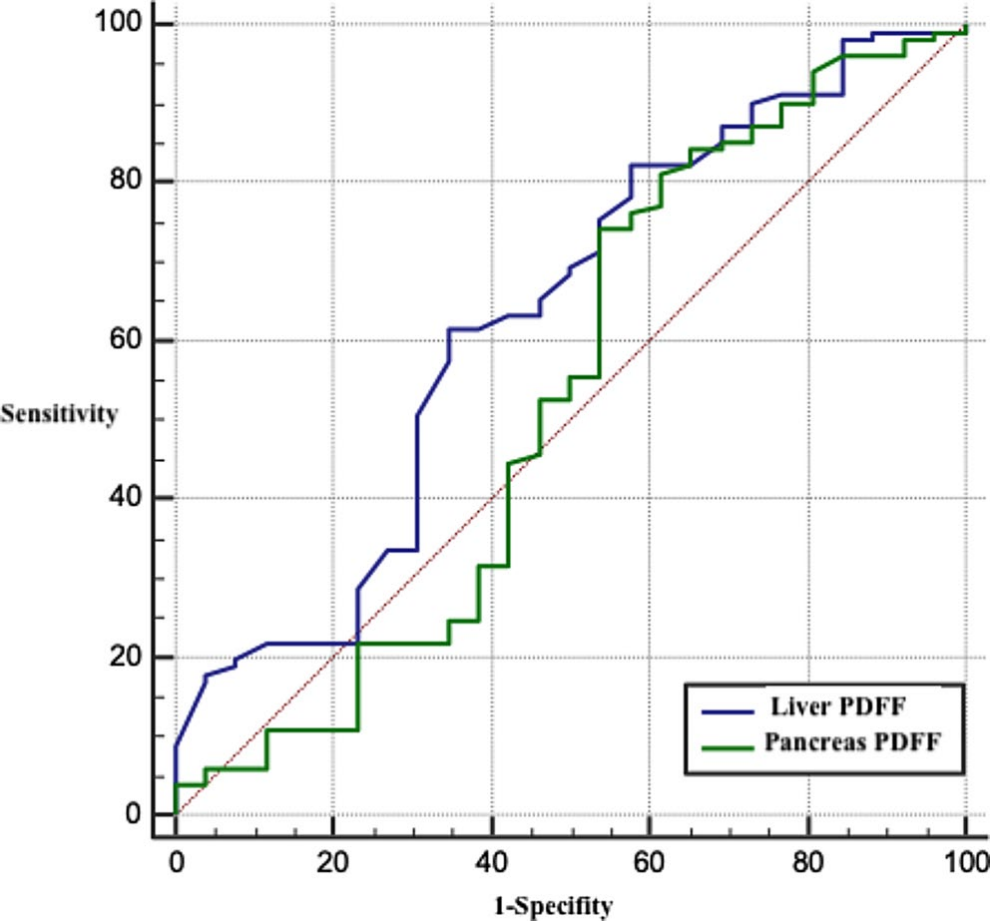
ROC curves of pancreatic and liver FF levels for the diagnosis of biliary pancreatitis

The roles of pancreatic and liver fat fractions in the etiology of pancreatitis were assessed using ROC analyses. Liver PDFF levels proved crucial in diagnosing biliary pancreatitis (AUC = 0.63, *p* = 0.042)(Fig. 5). Specifically, a liver PDFF level below 4.5 yielded a sensitivity of 61.3%, a specificity of 65.3%, positive predictive value of 87.3% and a negative predictive value of 30.4% for biliary pancreatitis.

## Discussion

Acute pancreatitis results from the premature activation of digestive enzymes within the pancreas, leading to tissue autodigestion. Normally, enzymes like trypsinogen remain inactive and are activated by enterokinase in the intestine to prevent self-digestion. However, pathological stimuli such as gallstones, alcohol abuse, obesity, and metabolic disorders like MASLD and NAFPD disrupt this balance, triggering the premature conversion of trypsinogen to trypsin. Cathepsin B directly activates trypsinogen, elevating trypsin levels and exacerbating tissue damage [25]. Molecular pathways contributing to this process include dysregulated calcium signaling, which causes mitochondrial dysfunction and oxidative stress, as well as the activation of inflammatory cytokines such as TNF-α and IL-6, which amplify the inflammatory response [26, 27]. Additionally, adipose tissue secretes proinflammatory adipokines and cytokines, further enhancing the systemic inflammatory response in AP [7].

Acute pancreatitis often has a mild and self-limiting course, but in 10–20% of cases, it can be severe, and mortality rises among these patient group [28]. The accumulation of neutral fat in the liver cells accompanied by progressive liver inflammation is considered a hepatic metabolic disorder [29]. Our findings indicate that acute pancreatitis is more severe in patients with MASLD. Liver PDFF levels were higher in cases of non-biliary pancreatitis, and although non-biliary causes were more commonly observed among patients with MASLD, a statistically borderline but not significant relationship was found (*p* = 0.051). Regarding NAFPD, no significant relationship or correlation was found between disease severity and diverse etiologies.

Yoon et al. [30] investigated the effect of steatotic liver disease (SLD) on the severity of acute pancreatitis using non-contrast CT in 200 patients. They found that the prevalence of SLD was significantly higher in patients with moderate and severe AP. Wu et al. [4] evaluated the effect of MASLD on the severity of AP using the revised Atlanta classification in a study with 656 patients. They reported that the severity of disease was higher in the moderate-severe MASLD group than in the mild group. Liu et al. [31] investigated the effect of SLD on AP severity in 189 patients and found that Acute Physiology and Chronic Health Evaluation II (APACHE-II) and mCTSI scores were higher in patients with SLD. Also, SLD was observed more frequently in non-biliary pancreatitis. Our findings are consistent with previous studies regarding the impact of MASLD and AP severity. In addition, a liver PDFF value above 3.9% has a diagnostic role in severe pancreatitis. This cut-off value indicates that severe pancreatitis may also develop in patients without MASLD.

Evaluated pancreatic steatosis using the IDEAL MRI method and reported that there was no significant difference in pancreatic PDFF levels between mild and moderate-severe pancreatitis groups according to the MR severity index (*p* > 0.050). Xie et al. [32] compared pancreas CT Hounsfield Unit (HU) levels with AP severity and found that systemic inflammatory response syndrome, APACHE-II, and Ranson scores increased correlatively as HU levels decreased due to pancreatic steatosis. However, the modified CT Severity Index (mCTSI) score did not show a significant correlation. In our study, no significant pancreatic PDFF correlation was found between mild, moderate, and severe AP groups. Furthermore, there were no differences in RAC, CTSI, or mCTSI scores between the groups with and without NAFPD. NAFPD is a newly recognised entity and may be a component of metabolic syndrome. Research indicates that NAFPD may adversely affect the prognosis of acute pancreatitis, particularly in patients with comorbidities. Methods and threshold values for measuring pancreatic steatosis vary across studies, potentially contributing to inconsistent results. In this study, we employed the IDEAL-IQ MRI sequence and established a threshold value of 10.4% for diagnosing NAFPD. This variation in methodology and thresholds likely contributes to differences in study results. Additionally, variations in patient populations and genetic and metabolic factors may influence findings.

Also, included 72 AP patients and 82 healthy controls in their study and reported that 5.1% pancreatic PDFF level had 100% sensitivity and 90.2% specificity in diagnosing AP. As there was no healthy control group in our study, we cannot evaluate the diagnostic performance of IDEAL PDFF. However, our ROC analysis demonstrated that a liver PDFF level of less than 4.5% supported the diagnosis of biliary pancreatitis when differentiating between biliary and non-biliary pancreatitis. The presence of hypertriglyceridemic patients in non-biliary pancreatitis and the primary role of MASLD in the etiology may have contributed to the observed differences in liver PDFF levels. In addition, in patients with high liver PDFF but also gallstones, diagnosing non-biliary pancreatitis may be misleading, and vice versa. In this case, it may be appropriate to use PDFF levels as a supportive finding.

Studies using various imaging modalities have reported that acute pancreatitis patients with MASLD and NAFPD have increased local and systemic complications, persistent organ failure, and development of systemic inflammatory response syndrome, and consequently higher mortality rates [4, 5, 30, 31, 32]. In our study, we found no significant difference in both liver and pancreas PDFF levels between patients with and without acute peripancreatic fluid collection, a local complication. Statistical evaluation could not be performed due to the low number of other complications.

This study has several limitations. Although our post-hoc power analysis confirmed adequate power for key comparisons, certain subgroup analyses, such as the comparison between mild AP and moderately severe to severe AP, exhibited insufficient power (~ 0.68). This may have limited our ability to detect significant associations, increasing the potential for Type II errors and suggesting that non-significant results should be interpreted with caution. Additionally, the number of patients with severe acute pancreatitis was lower than in other groups, primarily due to their poor general condition, making them unsuitable for MR-MRCP imaging. Furthermore, patients eligible for MR-MRCP imaging were more prone to motion artifacts, leading to unreliable measurements and potential biases. Another limitation is the absence of baseline pancreatic fat fraction data prior to inflammation, which prevented assessment of acute pancreatic inflammation’s effect on PDFF measurements. Lastly, as a single-center retrospective study, the generalizability of our findings is limited, and additional biases may be present.

## Conclusion

Patients with MASLD may be at an increased risk of developing complications and experiencing a more severe course of acute pancreatitis. Liver fat fractions, measured using the IDEAL MRI, could serve as supportive biomarkers for radiologists to predict the likelihood of severe acute pancreatitis and to differentiate between biliary and non-biliary etiologies. However, the limited statistical power observed in subgroup analyses highlights the necessity for larger-scale studies to validate these associations and improve their applicability across diverse populations. Further research is essential to fully elucidate the diagnostic value of the IDEAL method in clinical settings.

## Electronic supplementary material

Below is the link to the electronic supplementary material.

Supplementary Material 1

## Data Availability

No datasets were generated or analysed during the current study.

## Declarations

### Meeting presentation

Presented at the 44th National Congress of Radiology, Antalya, Turkey, November 21–25, 2023.
