# Supplementary material for: Liver and pancreatic fat fractions as predictors of disease severity in acute pancreatitis: an MRI IDEAL-IQ study
Source: Abdom Radiol (NY). 2025 Jan 30;50(8):3734–43. doi: 10.1007/s00261-025-04809-y (PMC12267334; doi:10.1007/s00261-025-04809-y)
Supplement: Supplementary file 1 — Supplementary Material 1 [file 261_2025_4809_MOESM1_ESM.pptx]

## Slide 1
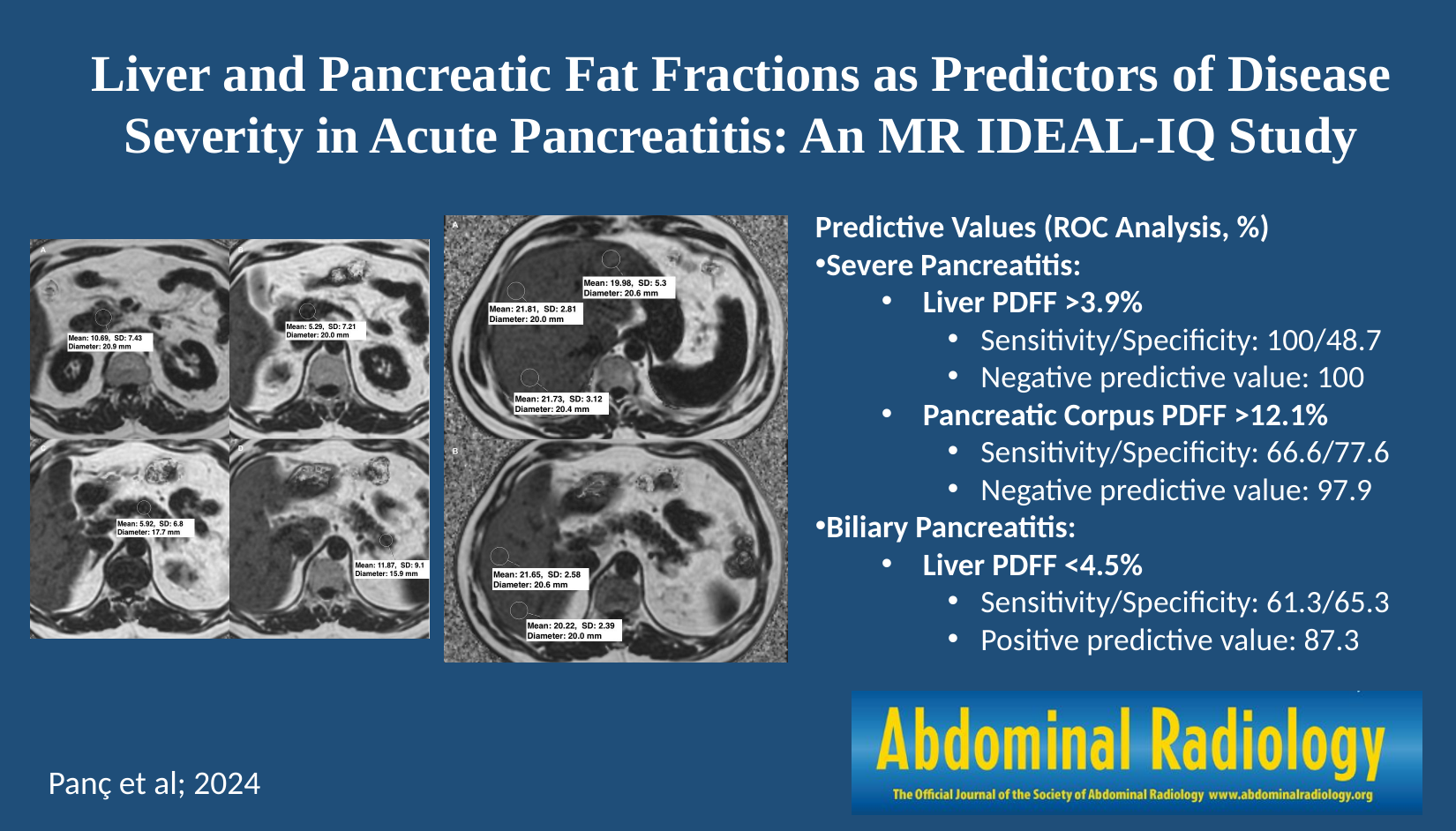

Liver and Pancreatic Fat Fractions as Predictors of Disease Severity in Acute Pancreatitis: An MR IDEAL-IQ Study
Predictive Values (ROC Analysis, %)
Severe Pancreatitis:
Liver PDFF >3.9%
Sensitivity/Specificity: 100/48.7
Negative predictive value: 100
Pancreatic Corpus PDFF >12.1%
Sensitivity/Specificity: 66.6/77.6
Negative predictive value: 97.9
Biliary Pancreatitis:
Liver PDFF <4.5%
Sensitivity/Specificity: 61.3/65.3
Positive predictive value: 87.3
Panç et al; 2024
